# Surgical treatment of congenital anomalies of the aortic arch : Long-term results

**DOI:** 10.1186/1749-8090-10-S1-A66

**Published:** 2015-12-16

**Authors:** Fabio Cuttone, Romain Amadieu, Géraldine Labouret, Lionel Berthomieu, Sophie Breinig, Sebastien Hascoet, Khaled Hadeed, Daniel Roux, Yves Dulac, Philippe Acar, Bertrand Leobon

**Affiliations:** 1 Department of Cardiac Surgery, University Hospital (CHU), Toulouse, France; 2 Department of Cardiology, University Hospital (CHU), Toulouse, France; 3 Department of Pediatric Pneumology and Allergology, University Hospital (CHU), Toulouse, France; 4 Department of Pediatric Intensive Care Unit, University Hospital (CHU), Toulouse, France

## Background/Introduction

Congenital anomalies of the aortic arch gather a wide spectrum of malformations concerning the aortic arch or some of its branches and the pulmonary artery. These malformations are rare (1 in 1000 live births) and sometimes responsible of complete (vascular ring) or partial (vascular sling) encirclement of the oesophagus, trachea or bronchi. In cases of symptomatic airways obstruction or oesophagus compression the surgical treatment is mandatory and curative.

In this paper we describe the experience of Children's Hospital of Toulouse on the surgical treatment of congenital anomalies of aortic arch.

## Aims/Objectives

To evaluate the results and the clinical outcome of reconstructive surgery in patients affected by symptomatic vascular ring.

## Method

Observational retrospective monocentric study including all the patients with a congenital anomaly of the aortic arch submitted to surgical treatment since May 2010, at the Children's Hospital of Toulouse.

## Results

Between May 2010 and March 2015, 10 patients (3 girls and 8 boys) underwent surgical treatment for vascular ring responsible for a tracheo-esophageal compression: 9 double aortic arch including 7 right dominant arches, 1 left dominant, 1 balanced disposition and 1 child with a Neuhauser anomaly.

The average age and weight were respectively 2.5 ± 1.5 years and 12.9 ± 3.7 kg. Surgical correction was carried out mainly by postero-lateral thoracotomy and was performed without complication. The mean duration of mechanical ventilation was 6 ± 7 hours and the average length of stay in intensive care was 1.7 ± 0.7 days. The average hospital stay was 4.8 ± 1.2 days. All patients are alive at the time of the last follow-up and asymptomatic with normal growth.

## Discussion/Conclusion

In this series, the surgical treatment of aortic arch anomalies was curative in all cases without associated morbidity and with good functional midterm results. The diagnosis is often delayed and more frequently suspected on the basis of respiratory symptoms.

## Consent

Written informed consent was obtained from the patient for publication of this abstract and any accompanying images. A copy of the written consent is available for review by the Editor of this journal.

